# Normative data for pituitary size and volume in the general population between 50 and 66 years

**DOI:** 10.1007/s11102-021-01150-7

**Published:** 2021-05-10

**Authors:** Erik Magnus Berntsen, Matias Daleng Haukedal, Asta Kristine Håberg

**Affiliations:** 1 Department of Radiology and Nuclear Medicine, St. Olavs Hospital, Trondheim University Hospital, Trondheim, Norway; 2 Department of Circulation and Medical Imaging, Faculty of Medicine and Health Sciences, Norwegian University of Science and Technology, Trondheim, Norway; 3 Department of Neuromedicine and Movement Sciences, Norwegian University of Science and Technology, Trondheim, Norway

**Keywords:** Pituitary, Size, Volume, Normal, Radiology

## Abstract

**Purpose:**

The main aim of this study was to provide normative data for pituitary height and volume in persons between 50 and 66 years in the general population. The secondary aim was to establish a convenient surrogate marker of pituitary size for use in routine radiological practice.

**Methods:**

From a geographically defined prospective healthy study, 1006 participants between 50 and 66 years had a brain MRI, of which 988 (519 women) were included in this study. We measured the mid-sagittal height, max-sagittal height and total volume of the anterior pituitary lobe based on T1-weighted 3D MRI images.

**Results:**

Both the mean mid-sagittal and max-sagittal pituitary height were significantly larger in women compared to men, with 4.9 ± 1.7 mm versus 4.4 ± 1.4 mm (p < .001) for the mean mid-sagittal height and 6.8 ± 1.2 mm versus 6.1 ± 1.1 mm (p < 0.001) for the mean max-sagittal height. The mean anterior pituitary lobe volume was also significantly larger in women than in men (494 ± 138 mm^3^ vs. 405 ± 118 mm^3^) (p < 0.001). There were no significant differences in these pituitary sagittal heights nor volume in either sex between the age groups 50–54, 55–59 and 60–66 years. The 95th percentile for mid-sagittal height, max-sagittal height and pituitary volume was 7.7 mm, 8.6 mm and 851 mm^3^ for women and 6.6 mm, 7.8 mm and 610 mm^3^ for men.

**Conclusion:**

This study show that women have a larger pituitary gland than men in the age group between 50 and 66 years and provides normative data for pituitary size estimates which can be used for clinical diagnostic purposes as well as future research.

## Introduction

The normal development of the pituitary gland is dependent on fluctuating neuroendocrine changes throughout life, thus the pituitary height and volume naturally varies with age and sex across the lifespan [1]. The majority of Magnetic Resonance Imaging (MRI) studies conducted on the normal physiological development of adolescence and adult pituitary size agrees that the pituitary size reaches its peak sometime during the 2nd or 3rd decade of life, and later declines in both men and women [1–5]. There is, however, conflicting results in the literature when it comes to persons above 50 years. Several studies report that women have larger pituitary glands than men in the 6th and 7th decade [1, 3, 4, 6, 7]. Some studies even report a tendency of increasing size of the pituitary in women above 50 years. It has been suggested that this could be due to post-menopausal increase in levels of gonadotropic hormones following loss of negative feedback from gonadal steroids [1, 4, 5]. In contrast, other studies report men having larger pituitary than women in this age group and one study even report an increase in pituitary size in men [2, 8]. To resolve these conflicting results and establish normative data we manually measured the pituitary volume using volumetric segmentation in a large cohort of 1006 individuals between 50 and 66 years from a general population. We also wanted to establish a measure of pituitary size convenient for routine radiological use. Volumetric segmentation of the pituitary is a manual and time-consuming process, while measuring the sagittal height is easier and could be a good surrogate marker for pituitary size. Most of the previous literature uses this sagittal height but at the same time states that there are large variations in pituitary morphology, e.g., the upper surface can be concave, flat or convex [1, 5–7, 9]. In cases with a concave surface, the largest height of the pituitary will not be in the mid-sagittal plane but lateral to it, hence the mid-sagittal height will not necessarily reflect the largest height of the gland. To overcome this, we sought to find an alternative height measurement correlating better with the measured volume of the pituitary.

The main aim of this study was to provide normative data for pituitary height and volume in persons between 50–66 years in the general population. The secondary aim was to establish a convenient surrogate marker of pituitary size for use in routine clinical radiological practice.

## Material and methods

**Fig. 1:**
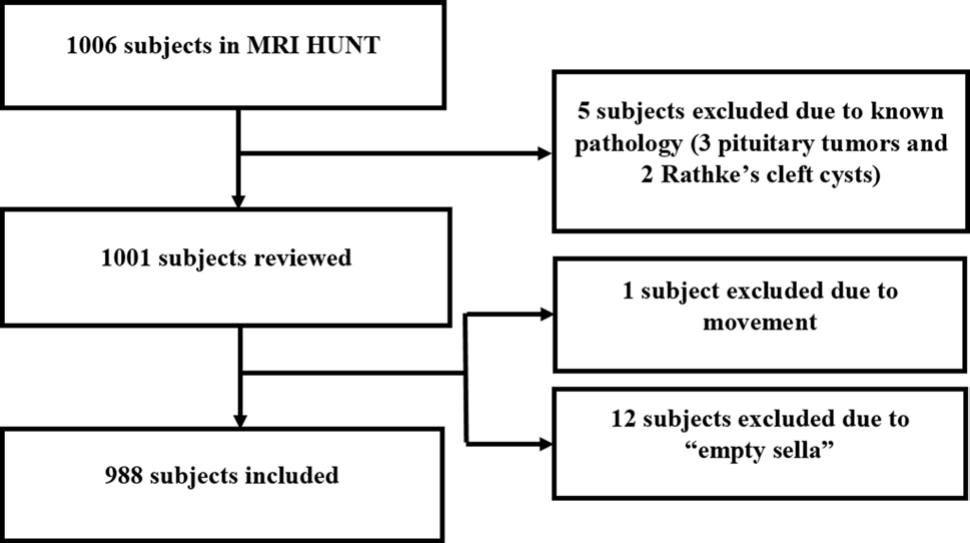
Flow chart of included subjects

During the Nord-Trøndelag Health Study (HUNT), which is a large geographically defined prospective health study on inhabitants above 13 years in the north part of the county of Trøndelag in Norway, 1006 HUNT participants between 50 and 66 years underwent a brain MRI-examination (HUNT MRI) in the period from 2007 to 2009 [10]. We considered all 1006 MRI-examinations for inclusion in the current study (Fig. 1). The HUNT MRI study and the current study were approved by the HUNT study board of directors, and the Regional Committee for Medical and Health Research Ethics in Central Norway (2011/456 and 2018/2231). Both the HUNT MRI study and the current study adhered to ethical standards.

The HUNT MRI scans were acquired on the same 1.5 T General Electric Signa HDx MRI-scanner and included a non-contrast enhanced sagittal 3D T_1_ weighted volume with 1.20 mm slice thickness and in-plane resolution of 0.975 × 0.975 mm^2^ which was used in this study. Two experienced neuroradiologists reviewed the MRI scans prospectively during data collection, and a paper on incidental intracranial findings is published [10]. In three of the subjects a pituitary tumour was discovered, and in two a Rathke’s cleft cyst was reported. These participants were excluded from further analysis. This left us with 1001 subjects with presumably normal pituitaries. All 1001 MRI-scans were reviewed and measurements performed by one of the authors (MH) after receiving extensive training in MRI-reading from a board certified neuroradiologist (EMB). In all difficult cases the neuroradiologist was consulted and guided the measurements.

### MRI imaging measurements

**Fig. 2:**
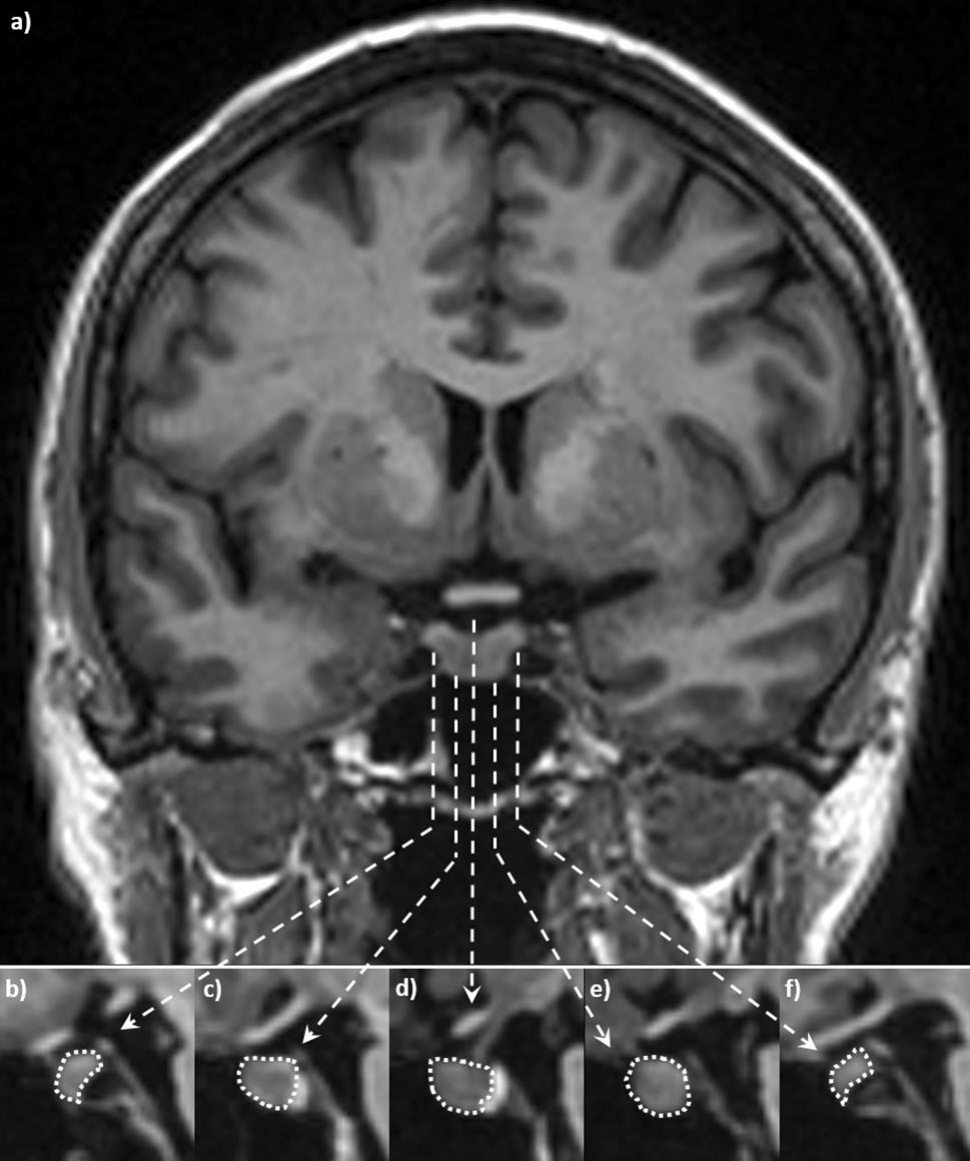
Coronal (**a**) and five representative sagittal (**b–f**) T1 weighted images of one subject’s pituitary gland. The volume of the anterior pituitary lobe was calculated by segmenting the area of the anterior lobe in all sagittal slices where it was visible, and then the sum of the areas was multiplied by the slice thickness of 1.20 mm

Volumetric segmentation of the anterior pituitary lobe was performed in each subject by manually marking the outline of the anterior pituitary lobe on all sagittal slices using the software used for clinical radiological reading at our hospital which automatically calculates the area marked (Sectra Workstation, IDS7, version 19.1). To calculate the volume of the segmentations, all areas measured were added together and multiplied by the slice thickness (Volume = (Σ_(Area anterior lobe)_ × slice thickness)) (Fig. 2). The posterior pituitary lobe volume was not included in this study.

**Fig. 3:**
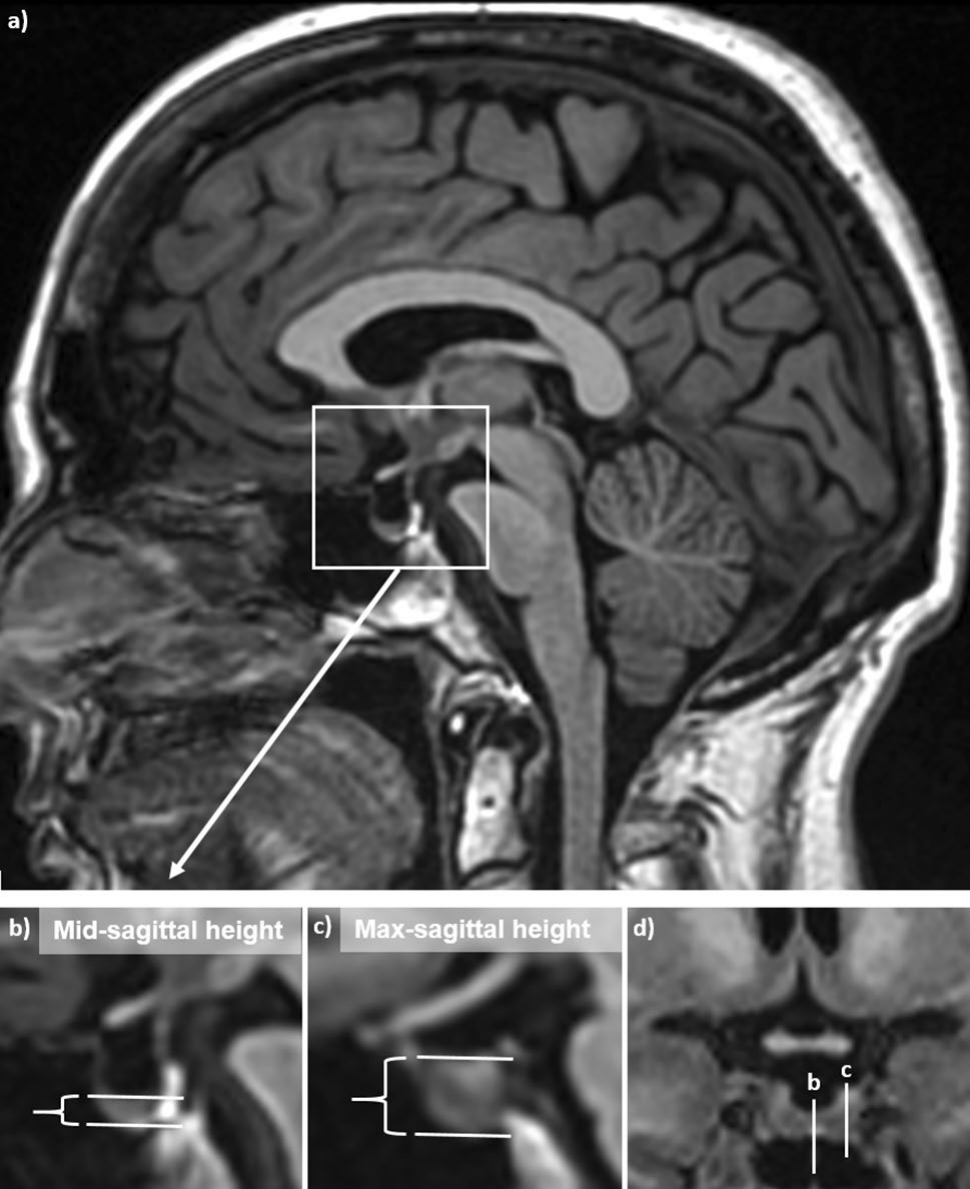
Mid-sagittal T1 weighted image of one subject (**a**) with magnified view showing the mid-sagittal height (**b**) of the anterior pituitary lobe measured to 3.9 mm, compared to the max-sagittal height measured to 8.7 mm. The coronal image (**d**) shows the sagittal planes of image (**b**) and (**c**)

The mid-sagittal height of the pituitary was measured as the craniocaudal height of the pituitary from its caudal border to the insertion point of the pituitary stalk in the mid-sagittal plane defined as the plane where the anterior lobe, posterior lobe and the pituitary stalk were visible. We also measured the craniocaudal height of the pituitary in the sagittal plane with the largest craniocaudal height, hereafter referred to as the max-sagittal height (Fig. 3).

Those cases were there was no measurable anterior pituitary tissue were classified as so-called “empty sella”, i.e., a sella turcica filled with cerebrospinal fluid and no visible pituitary tissue. Thus, those subjects who could have been categorized as “partial empty sella” were included and measured.

### Statistical analysis

All statistical analysis was performed using SPSS version 25 (Armonk, NY, US). Mean values for mid-sagittal height, max-sagittal height and volume were calculated in men and women separately and in age-defined subgroups. Differences between sexes were investigated using two tailed independent-samples t-test, while differences between subgroups (age-intervals 50–54 years, 55–59 years and 60–66 years) were investigated separate for each sex using one-way ANOVA-analysis. Any difference between the two sagittal measures of sagittal pituitary height was investigated using paired samples t-test, while any correlation to volume was investigated using Pearson´s correlation coefficient. P-values < 0.05 was considered statistically significant for both the t-tests and ANOVAs. The 1st, 5th, 50th, 95th and 99th percentiles for mid-sagittal height, max-sagittal height and volume were calculated separately for each sex.

## Results

The MRIs from the 1001 subjects with presumed normal pituitary were reviewed. Subsequently one participant was excluded due to insufficient image quality following head movement, and 12 participants were excluded due to so-called “empty sella”. The remaining 988 subjects were included in the analysis, of which 519 were women (mean age 58.2 years) and 469 men (mean age 58.7 years).

The mean pituitary volume for men was 405 ± 118 mm^3^ (range 116–896 mm^3^) and 494 ± 138 mm^3^ for women (range 114–1089 mm^3^), constituting a significant difference (p < 0.001). The mean mid-sagittal pituitary height for men was 4.4 ± 1.4 mm (range 0.7–8.8 mm) and for women 4.9 ± 1.7 mm (range 0.0–11.2 mm), constituting a significant difference (p < 0.001). The mean max-sagittal height for men was 6.1 ± 1.1 mm (range 3.0–9.8 mm) and 6.8 ± 1.2 mm (range 2.0–11.2 mm) for women, constituting a significant difference (p < 0.001).

**Fig. 4:**
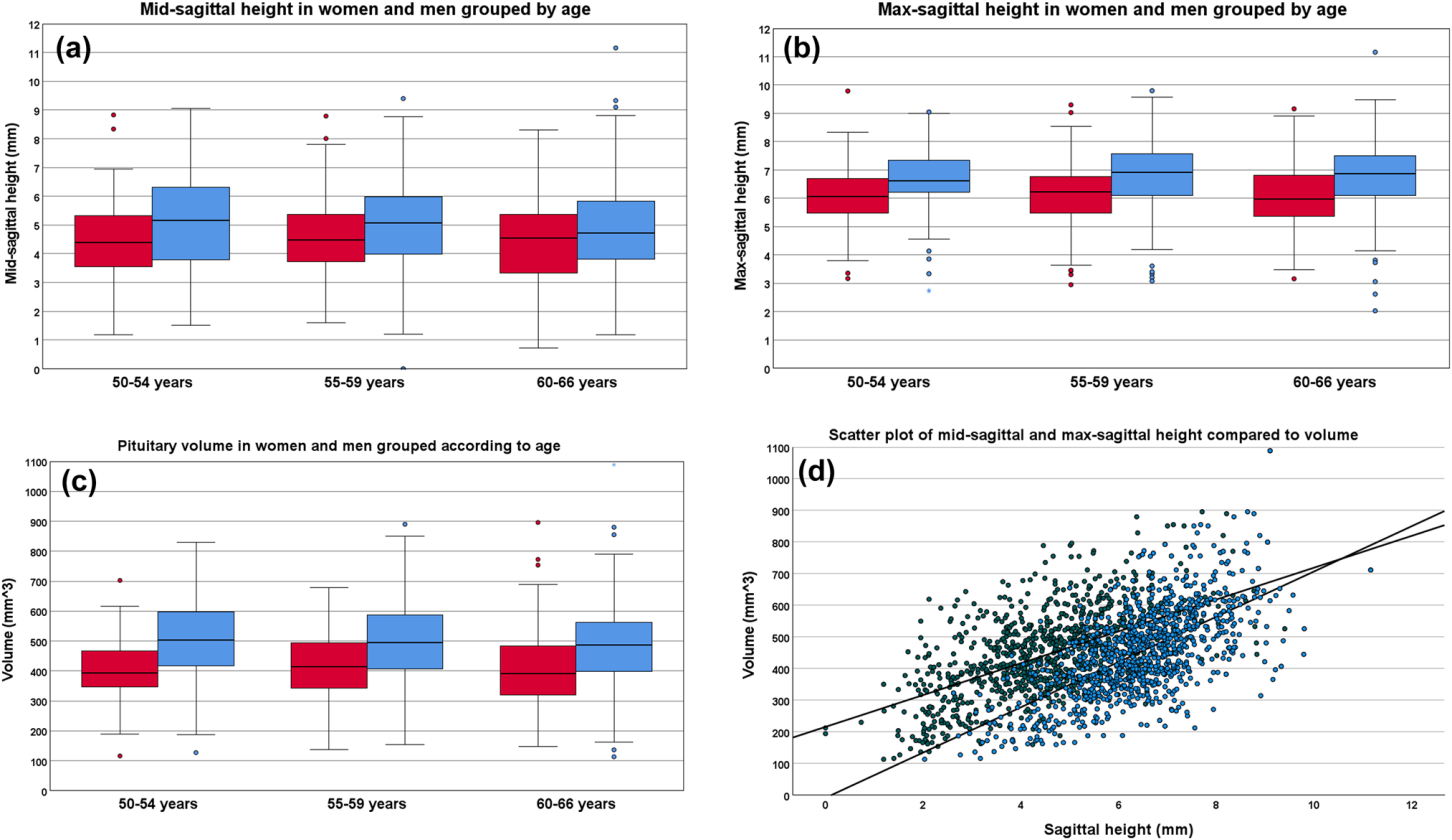
Boxplots of (**a**) Mid-sagittal height, (**b**) Max-sagittal height, and (**c**) Volume of the anterior pituitary lobe in men and women grouped by age, with statistical significant differences marked with an asterisk (*). Red boxes represent men and blue boxes women. The dark lines within each boxes represent the median, while the boxes lower and upper boundaries represent the 25th and 75th percentile, respectively, also called the interquartile range (IQR) and containing the middle 50% of the measurements. Each whisker extends to 1.5 times the IQR. Circles represent outliers. In (**d**) a scatterplot of the mid-sagittal and max-sagittal height compared to volume showing the difference in distribution and correlation to volume, with a stronger correlation for the max-sagittal height

Subjects were further categorized into three age groups: 50–54 years, 55–59 years, and 60–66 years to investigate whether there were any significant changes in pituitary size across these age groups in each sex (Table 1; Fig. 4a–c). There were no statistically significant differences for neither men nor women with respect to mid-sagittal height, max-sagittal height or pituitary volume across these age groups according to the ANOVA-analysis (Fig. 4a–c).

**Table 1:** Mean values of pituitary mid-sagittal height, max-sagittal height and anterior pituitary lobe volume in men and women in the different age groups

Age group	Sex	Mid-sagittal height in mm (mean ± SD)	Max-sagittal height in mm (mean ± SD)	Anterior pituitary lobe volume in mm^3^ (mean ± SD)
50–54	Men (n = 90)	4.41 (± 1.52)	6.10 (± 1.04)	400 (± 100)
	Women (n = 119)	5.06 (± 1.63)	6.66 (± 1.17)	505 (± 137)
55–59	Men (n = 159)	4.47 (± 1.34)	6.11 (± 1.10)	415 (± 116)
	Women (n = 187)	4.90 (± 1.67)	6.78 (± 1.24)	494 (± 143)
60–66	Men (n = 220)	4.40 (± 1.43)	6.03 (± 1.12)	398 (± 126)
	Women (n = 213)	4.85 (± 1.64)	6.78 (± 1.25)	489 (± 135)
Total	Men (n = 469)	4.43 (± 1.42)	6.07 (± 1.10)	405 (± 118)
	Women (n = 519)	4.92 (± 1.65)	6.75 (± 1.23)	494 (± 138)

*n* number of subjects, *SD* standard deviation

There was a statistically significant difference between mid-sagittal and max-sagittal height of 1.7 ± 1.4 mm (p < 0.001) according to the paired samples t-test. There was a statistically significant strong correlation between mid-sagittal height and pituitary volume for the sexes combined with r(986) = 0.58 (p < 0.01), but an even stronger correlation between the max-sagittal height and pituitary volume with r(986) = 0.64, p < 0.01 (Pearson´s correlation) (Fig. 4d).

In order to provide normative data for use in radiological practice we calculated the 1st, 5th, 50th, 95th and 99th percentile for each sex (Table 2). The 95th percentile for mid-sagittal height, max-sagittal height and pituitary volume was 7.7 mm, 8.6 mm and 736 mm^3^ for women and 6.7 mm, 7.8 mm and 610 mm^3^ for men.

**Table 2:** Normative data for sagittal heights and volume of the anterior pituitary lobe given as the 1st, 5th, 50th, 95th and 99th percentile for each sex

	Men (n = 469)					Women (n = 519)				
	1 p.	5 p.	50 p.	95 p.	99 p.	1 p.	5 p.	50 p.	95 p.	99 p.
Mid-sagittal height (mm)	1.46	2.04	4.50	6.66	8.14	1.54	2.11	4.96	7.70	9.01
Max-sagittal height (mm)	3.35	4.06	6.12	7.80	8.95	3.06	4.48	6.83	8.64	9.36
Volume (mm^3)	162	209	398	610	692	163	268	494	736	851

The 50th percentile is equal to the median

*n* number of subjects. *p.* percentile

## Discussion

Normative data for pituitary size in the general population is warranted, as there is a lack of consensus in the literature with regard to size and differences between the sexes for persons above 50 years. Clarifying these inconsistencies is of importance for medical professions dealing with pituitary disease, in order to provide better guidance for clinical decisions. A convenient surrogate marker for the pituitary size is of great radiological interest in order to readily detect and separate normal from pathological pituitary gland sizes. Our study was successful in obtaining normative data in a large representative cohort from a general population between 50 and 66 years [11], as well as providing 95th and 99th percentiles for pituitary volume and sagittal heights for use in radiological practice.

Our results showed that the pituitary gland is significantly larger in women, being in accordance with most previous studies (Table 3) [1, 3, 5–7]. Furthermore, we found no significant difference in pituitary size across different age groups. These findings were consistent for all three measurement methods used in this study. As our study has far greater sample size and higher spatial resolution than previous studies, and is based on gold standard manual segmentation, we believe our results are more accurate than those previous reported.

**Table 3:** Summary of previous studies on pituitary size with reported mean values of pituitary mid-sagittal height and volume in men and women in different age groups, compared to our study

Study	Sex	Age	n	Mid-sagittal height in mm ± SD	Pituitary volume in mm^3^ ± SD
	Men	50–59	26	4.4 ± 1.6	–
Suzuki et al		60–69	25	4.2 ± 1.2	–
1990	Women	50–59	28	4.6 ± 1.7	–
		60–69	18	4.9 ± 1.6	–
	Men	50–59	2	3.8 ± 1.3	–
Doraiswamy et al		60–69	5	4.6 ± 1.0	–
1992	Women	50–59	6	4.1 ± 0.9	–
		60–69	8	5.7 ± 1.7	–
	Men	50–59	117	4.80 ± 1.03	–
Tsunoda et al		60–69	134	4.78 ± 1.02	–
1997	Women	50–59	92	5.44 ± 1.18	–
		60–69	137	4.88 ± 1.07	–
	Men	51–60	13	6.4 ± 0.5	–
Denk et al		≥ 61	11	5.8 ± 0.6	–
1999	Women	51–60	13	4.8 ± 0.5	–
		≥ 61	9	5.0 ± 0.4	–
	Men	51–60	16	6.3 ± 1.4	335 ± 170
Ibinaiye et al		61–70	3	5.1 ± 2.9	217 ± 201
2015	Women	51–60	5	6.4 ± 1.7	298 ± 49
		61–70	2	5.0 ± 0.4	292 ± 124
	Men	44–54	5	4.85 ± 0.01	–
Mohieldin et al		55–65	2	4.47 ± 0.00	–
2016	Women	44–54	4	5.63 ± 0.63	–
		55–65	4	4.75 ± 0.17	–
Yadav et al	Men	≥ 50	-	6.0 ± 1.6	410 ± 168
2017	Women	≥ 50	-	6.7 ± 1.9	420 ± 174
Singh et al	Men	≥ 50	50	5.38 ± 1.21	329 ± 98
2018	Women	≥ 50	36	5.27 ± 1.14	345 ± 100
		50–54	90	4.41 ± 1.52	400 ± 100
	Men	55–59	159	4.47 ± 1.34	415 ± 116
Berntsen et al		60–66	220	4.40 ± 1.43	398 ± 126
2021		50–54	119	5.06 ± 1.63	505 ± 137
	Women	55–59	187	4.90 ± 1.67	494 ± 143
		60–66	213	4.85 ± 1.64	489 ± 135

*n* number of subjects. *SD* standard deviation

### Volume measurements

We found that women had statistically significant larger anterior pituitary lobe volume than men assessed with manual segmentation, being 494 ± 138 mm^3^ versus 405 ± 118 mm^3^, respectively. Only three previous studies have estimated the pituitary volume in healthy volunteers in the same age group as in this study (50–66 years). All of them have used formulas based on length, width and height of the pituitary, and not manual volumetric segmentation, which is the gold standard for radiological volume measurements. Yadav et al. used a formula assuming a sphere, giving women a slightly larger pituitary than men for subjects above 50 years (420 ± 174 mm^3^ vs. 410 ± 168 mm^3^, statistical comparison not performed, number of subjects in subgroups not stated) [5]. Ibinaiye et al. also used a formula assuming a sphere, surprisingly giving a smaller pituitary in women compared to men in their age group 51–60 years with 21 subjects (5 women) (298 ± 49 mm^3^ vs. 335 ± 170 mm^3^) [4]. However, in their age group 61–70 years with merely five subjects (two women), women had larger pituitaries than men (292 ± 124 mm^3^ vs. 217 ± 201 mm^3^). Singh et al. estimated the pituitary volume based on Di Chiro’s formula, which is slightly different from the volume of a sphere as the constant in the formula is 0.50 rather than 0.52, reporting a non-significant slightly larger pituitary for women compared to men (345 ± 100 mm^3^ vs. 329 ± 98 mm^3^, p = 0.47) [8, 12]. As we have a much larger sample size than the above studies (988 subjects vs. not-stated/26/86 subjects), thinner sagittal slices (1.2 mm vs. 5.0 mm/3.0 mm/not-stated) than the previous studies [4, 5, 8], and have performed manual segmentation of each pituitary, we believe our results are more accurate. Furthermore, as our volume estimates of the pituitary volume is clearly larger, we speculate that calculating pituitary volume indirectly by using a formula in combination with a lower image resolution underestimates the anterior pituitary volume.

### Sagittal height measurements

We found that women had statistically significant larger pituitary height than men assessed with both the mid-sagittal and max-sagittal measurement, being 4.9 ± 1.7 mm versus 4.4 ± 1.4 mm and 6.8 ± 1.2 mm versus 6.1 ± 1.1 mm, respectively. Only the mid-sagittal height has been investigated in previous studies. Suzuki et al. used a mid-sagittal measurement of the pituitary height and reported women having larger pituitaries than men in their cohort of 54 subjects (28 women) between 50 and 59 years (4.6 ± 1.7 mm vs. 4.4 ± 1.6 mm), as well as in their cohort of 43 subjects (18 women) between 60 and 69 years (4.9 ± 1.6 mm vs. 4.2 ± 1.2 mm) (study population not clearly stated) [6]. No statistical comparisons were performed, but their results are similar to ours. Tsunoda et al. also used a mid-sagittal measurement and reported significantly larger in women than men (5.4 ± 1.2 mm vs. 4.8 ± 1.03 mm) in their cohort of 209 patients (92 women) between 50 and 59 years from a Japanese inpatients population that underwent routine brain MRI [1]. They did however not find a significant difference in their cohort of 271 patients (137 women) between 60 and 69 years (4.88 ± 1.07 mm vs. 4.78 ± 1.02 mm), which is in contrast to our results. The studies of Suzuki et al. and Tsunoda et al. were published in 1990 and 1997 and had voxel-sizes of 1.56 × 0.78 × 5.0 mm^3^ and 1.56 × 0.78 × 9.0 mm^3^, which compared to our 1.25 × 1.25 × 1.20 mm^3^ are quite large. Thus, our study has higher image resolution giving estimates that are more precise. The studies from Suzuki et al. and Tsunoda et al. are the largest previously published in the literature, with 97 subjects and 468 patients, but still substantial smaller than ours consisting of 988 participants. In addition, some smaller studies also report the same sex differences using the mid-sagittal height of the pituitary gland. Mohielden et al. found a larger sagittal pituitary height for women than men in healthy subjects from a Sudanese population in their subgroup of 9 subjects (4 women) in the age group 44–54 years (5.6 ± 0.6 mm vs. 4.9 ± 0.0 mm), as well as in their subgroup of 6 subjects (4 women) in the age group 55–65 years [3]. Doraiswamy et al. used the mid-sagittal height from healthy volunteers from the US and reported that the pituitary was larger in women compared to men in their subgroup of 8 subjects (6 women) in the age group 50–59 years (4.1 ± 0.9 mm vs. 3.8 ± 1.3 mm), as well as in their subgroup with 13 subjects (8 women) in the age group 60–69 years (5.7 ± 1.7 mm vs. 4.6 ± 1.0 mm) [7]. Yadav et al. also used a mid-sagittal measurement and reported women having a larger pituitary than men for Indian subjects between 50 and 80 years collected from a patient population (6.7 ± 1.9 mm vs. 6.0 ± 1.6 mm) (500 patients included in the whole study, but neither number of subjects in this sub-group stated nor statistical analyses performed) [5].

Some studies with much smaller groups do however report conflicting results compared to our study and the above literature. Ibinaiye et al. used a mid-sagittal measurement and reported women having a slightly larger pituitary than men for 21 subjects (5 women) collected from an Nigerian inpatient population in the age-group 51–60 years (6.4 ± 1.7 mm vs. 6.3 ± 1.4 mm), but men having a slightly higher pituitary comparing 5 subjects (2 women) in the age-group 61–70 years (5.0 ± 0.4 mm vs. 5.1 ± 2.9 mm) [4]. Given the small sample-size, the latter finding is likely not significant. Singh et al. used the mid-sagittal height and reported no significant difference between sexes for 86 subjects (36 women) above 50 years collected from an Indian inpatient population (5.3 ± 1.1 mm vs. 5.4 ± 1.2 mm, p = 0.66) [8]. Denk et al. used the mid-sagittal height and reported a smaller pituitary height in women than men in their cohort of 26 subjects (13 women) in the age group 51–60 (4.8 ± 0.5 mm vs. 6.4 ± 0.5 mm) (study performed in Turkey, population not stated) [2]. In their group above 60 years, with 20 subjects (9 women) they did however find that women had the largest pituitaries (5.0 ± 0.4 mm vs. 5.8 ± 0.6 mm). Pituitary height for men being larger than women is directly conflicting with our results and the two other studies with the largest sample size in the literature [1, 6]. Therefore, one has to question the validity of these contradicting results [2, 4, 8]. Each of these studies had smaller cohorts varying from 20 to 86 subjects, which probably have made their results more vulnerable to random variations. Furthermore, some of these studies recruited from an inpatient population or a population with suspected or confirmed disease, making these findings not representative for a general population.

One striking difference between the above studies is that they were performed in very different ethnic groups. Differences in pituitary size due to ethnicity have previously been hypothesised, but no proper study to investigate this has been undertaken [4]. It is however known that pituitary adenomas are more common in black Americans than other ethnic groups in America [13]. Our study participants were from a general population from one geographically defined region in Norway while the previous studies were performed in Japan, Sudan, United States, Nigeria, Turkey and India. If there is ethnical differences in pituitary size, this could be a contributing factor to the different results. Nevertheless, we believe that the most likely explanation for the differing results in these smaller studies is due to the smaller sample-size susceptible to random variation.

### Which estimate to use in radiological practice?

As volumetric segmentation is the gold standard for estimation of size and easy to perform, even though time consuming, we believe this is the method of choice if in need of a precise estimate. Our calculated 95th and 99th percentiles can be used as upper reference value being 736 mm^3^ and 851mm^3^ for women and 610 mm^3^ and 692 mm^3^ for men.

A simpler approach to estimating the pituitary size is to use the sagittal height of the pituitary, and we mean that the max-sagittal height should be used rather than the mid-sagittal height. The latter can be misleading due to asymmetry of the pituitary gland, causing the largest craniocaudal height to be off midline. This was clearly demonstrated in our study where the mid-sagittal and max-sagittal heights lower range was 0 mm and ≈ 2 mm, respectively, and further exemplified in Fig. 3. Furthermore, we found that there was a significant difference of 1.7 ± 1.4 mm between the two height measurements, and that the max-sagittal height had the strongest correlation to the volume estimate. Our 95th and 99th percentiles for max-sagittal height of 8.6 mm and 9.4 mm for women and 7.8 mm and 9.0 mm for men can be used as upper reference values.

The percentile values presented in Table 2 were calculated after the 12 subjects with no measurable pituitary tissue were excluded (e.g. so-called “empty sella”). If they were to be included with a sagittal heights and volume set to zero, the 1st percentile for sagittal height and volume would become zero in both women and men, but with only minor effect on the other presented percentiles in Table 2.

### Strengths and limitations

The major strength of our study is the large cohort of 988 subjects from the general population between 50 and 66 years, giving us between 90 and 220 subjects in each age subgroup, which by far is the largest study to date. The study with the second largest cohort is the one of Tsunoda et al. with 92 to 137 subjects in each subgroup [1]. Thus, our study has greater statistical power in finding statistically significant differences and less vulnerable to random variation. Most of the studies with conflicting results to our study had less than 20 subjects in each subgroup, and therefor clearly more vulnerable to random variation.

Another strength of our study is the higher image resolution than previous studies, enabling us to achieve more precise measurements. A slice thickness of 1.20 mm with submillimeter in-plane resolution allowed us to determine and distinguish between the mid-sagittal and the max-sagittal pituitary height. Furthermore, this allowed us to measure the volume of the pituitary more precisely than the previous studies which calculated the pituitary volume using formulas with height, width and length. However, one could argue that today’s golden standard is manual segmentation with even smaller voxels, as today’s MRI technology can produce submillimeter voxels in all dimensions.

Our study was performed on a 1.5 T MRI system, while many specialized pituitary centers use 3.0 T MRI systems nowadays, which provide better visualization of microadenomas and invasion of parasellar regions [14]. Using 3.0 T instead of 1.5 T MRI scanners in our study could have led to more precise estimates of the true pituitary heights and volumes, but taken into account our high in-plane resolution of 0.975 × 0.975 mm^2^ where all our manual measurements were performed, we do not believe it would change our results substantially. Therefore, we believe our results are possible to extrapolate also to 3.0 T MRI examinations. Furthermore, MRI at 1.5 T is still the more frequent than at 3.0 T, making our results generalizable to most MRI studies.

Ideally, several expert neuroradiologists should have performed manual segmentations of the pituitaries independently. However, given the large study population and time-consuming manual segmentations, this was deemed infeasible. Thus, the next best solution was chosen; a single medical student receiving extensive training in reading and manually segmenting the pituitary gland on the MRI scans of those participants that already had been read as normal by two experienced neuroradiologist. Furthermore, the student received guidance by the same board certified neuroradiologist segmenting all pituitaries the student had difficulties reading. We believe this is the most feasible way of performing manual segmentation in such large studies with more than a thousand participants, and that engaging expert neuroradiologist in such work might be a suboptimal use of expert resources.

As the data from this study was collected from a prospective health study in the general population, brain MRI was without gadolinium contrast. This is not a limitation when it comes to measuring the sagittal height and volume of the pituitary, but is a limitation when it comes to discovering pituitary tumours without mass effect such as microadenomas. The prevalence of macroadenomas discovered incidentally at MRI has been shown to be between 0.16 and 0.30%, which fits well with our finding of 3 pituitary tumours (one known from before and two incidentally discovered as part of the MRI HUNT study) [10, 15]. The prevalence of micro-incidentalomas discovered at MRI has traditionally been considered to be much higher and in the range of 10–38%, which however was not found in our study. Our low prevalence of incidental findings of 0.5% is however in line with a recent study by Kuo et al., where they applied more strict criteria to what was considered true incidental findings than in previous literature [16]. In their study, they found a prevalence of 0.1% incidentalomas (2 Rathke’s cleft cysts, 1 microadenoma and 1 macroadenoma in 3840 imaging studies (both MRI and CT)). Thus, our low prevalence of pituitary incidentalomas seems reasonable.

Participants included in our analyses were all presumably without pituitary disease. However, endocrinological evaluation of subjects was not performed, thus we cannot rule out that the subjects included could potentially have neuroendocrine disorders affecting the pituitary.

## Conclusion

The current study provides normative data for pituitary height and volume in persons between 50 and 66 years. We have found that women in this age group have larger a larger pituitary gland than men, and that there is no difference across age groups. Furthermore, when in need of a precise size estimate of the pituitary, volumetric segmentation should be preferred as this is superior to any geometrically based formula, with a 95th percentile value of 736 mm^3^ for women and 610 mm^3^ for men. Alternatively, when in need for rapid size estimate of the pituitary, the maximal sagittal height should be used with a 95th percentile of 8.6 mm for women and 7.8 mm for men.
